# Evaluation of the cognitive-motor performance of adults with Duchenne Muscular Dystrophy in a hand-related task

**DOI:** 10.1371/journal.pone.0228128

**Published:** 2020-01-31

**Authors:** Kostas Nizamis, Wouter Schutte, Jan J. Grutters, Jasper Goseling, Noortje H. M. Rijken, Bart F. J. M. Koopman

**Affiliations:** 1 Department of Biomechanical Engineering, Technical Medical Centre, University of Twente, Enschede, The Netherlands; 2 Stochastic Operations Group and the Data Science Group, University of Twente, Enschede, The Netherlands; 3 Department of Rehabilitation, Donders Institute for Brain, Cognition and Behaviour, Radboud University Medical Centre, GC Nijmegen, The Netherlands; Berner Fachhochschule, SWITZERLAND

## Abstract

Duchenne muscular Dystrophy (DMD) is a progressive degenerative muscle disease, affecting, among others, the upper extremities. Effective hand rehabilitation can improve the hand function of people with DMD. To reach this goal, we first need to gain more insight into the hand cognitive-motor performance of people with DMD. This is the first study employing a systematic analysis on multi-finger, cognitive-motor performance of people with DMD. For this purpose, we propose an active dynamic visuo-motor task. The task employed six visual stimuli, a subset of which was activated at each trial. The stimuli were activated with a frequency of 1, 2, 3 and 4 Hz. Eight healthy participants and three participants with DMD performed the task. Additionally, the healthy participants performed seven sessions, and we assessed the training effects. Task-related cognitive-motor performance was evaluated using information transfer rate (ITR) and perceived workload. Regarding ITR, healthy participants performed significantly better than DMD participants; however, this was more evident for trials involving more than three fingers. Workload showed no difference between the healthy and the DMD groups. Healthy participants significantly improved their performance during training. Our results suggest that hand rehabilitation of people with DMD should consider multi-finger dynamic training. However, additional research with more people with DMD is needed for further generalization of our conclusions.

## Introduction

Duchenne muscular dystrophy (DMD) is an X chromosome-linked recessive neuromuscular disease, affecting mainly males. It is diagnosed in childhood, affecting approximately 1:5000 births [1]. In 2013, the population of people with DMD in The Netherlands was 420 [2]. Duchenne is caused by mutations in the dystrophin gene that encodes the protein dystrophin, causing its absence or defect [3]. People with DMD suffer from progressive muscle weakness which leads to physical disability, high dependency on care-givers, and shortened life expectancy [4].

Due to advances in health care over the past few years, life expectancy has gradually increased, and currently people with DMD can reach the age of 40 [3]. The number of adults with DMD will grow substantially as future therapies, though not necessarily curing, will retard the disease, thus increasing the existing DMD population [5]. Although their lifespan has increased, their hand function remains limited, especially after the age of ten [6]. People with DMD may live longer with impaired hand function, and therefore will be unable to perform basic activities of daily living (ADL) for decades [7].

Still, the main clinically applied hand treatment for people with DMD includes physical therapy [5] and passive hand splints [8]. These aim at maintaining a large active range-of-motion (ROM) for the fingers and the wrist and slowing the development of contractures. Furthermore, studies investigating hand function in DMD concern the remaining hand ROM and strength [6, 9], but not dynamic finger performance.

People with DMD can benefit from active-hand-assistive technology that can provide continuous passive motion (CPM) or support their movement based on their intention [10]. In the Flextension Symbionics project [11], we are developing a wearable active-hand-assistive device with an intuitive control interface for people with DMD. To this end, we have studied the ability of people with DMD to control their hand during a visuo-motor task. The task has a motor component, related to the fingers pressing the buttons, and a cognitive component, related to the visuo-cognitive processing of visual stimuli. To the best knowledge of the authors, there is currently no detailed and systematic analysis of the cognitive-motor, multi-finger performance of people with DMD. Such a detailed analysis is needed to finally understand how, compared to healthy controls, people with DMD can perform. More insight into their hand cognitive-motor performance may enable the development of customized rehabilitation with the use of wearable active-assistive devices.

Inspired by the work of Klemmer et al. [12], we employed a visuo-motor task including the use of six fingers. Motor performance was measured via information transfer rate (ITR) [13]. To address the cognitive component of the task, we also measured the perceived workload imposed on the participant by the task [14]. The observation of both ITR and workload can show the optimal trade-off between cognitive and motor performance, as related to the task. In a previously conducted pilot we tested the feasibility of our protocol [15], and found that healthy participants and a participant with DMD were able to perform the visuo-motor task. However, the person with DMD showed a lower absolute performance in the task compared to healthy participants. The present study was conducted with eight other healthy participants who performed seven training sessions and two more people with DMD. In this study, we wanted to (I)compare the task performance of people with DMD to a healthy baseline performance, (II)analyze their motor performance together with their cognitive effort using a Pareto analysis and (III)study the effects of training on the task-related cognitive-motor performance in the healthy controls.

## Materials and methods

### Participants

The experiment was carried out by eight healthy adults (six male and two female), ranging from 19-24 years in age, without any hand-related impairment, and three adults with DMD (aged from 20-25). We included participants with different levels of hand function. Participant 1 (DMD 1, 20 yrs old) was able to functionally use his hand, and minimal contractures relevant to finger movement were observed. Participant 2 (DMD 2, 21 yrs old) was able to use his hands functionally, but he experienced a decrease in strength. Minimal contractures relevant to finger movement were observed. Lastly, participant 3 (DMD 3, 25 yrs old) was not able to use his hands for grabbing a pen and was experiencing strong fatigue during the use of his hand. Extensive contractures relevant to finger movement were observed. All participants were capable of clicking the buttons and performing the experiment. The Medical Ethics Committee of Twente decided that this study did not require an ethical approval regarding the healthy participants (Protocol number: K17-51). The study was conducted according to the ethical standards given in the Declaration of Helsinki in 1975, as revised in 2008. For the participants with DMD, the Medical Ethics Committee of Twente approved the study design, the experimental protocol, and the procedures (Protocol number: NL59061.044.16). Both healthy and DMD participants were informed via a letter and signed a consent form prior to the experiment.

### Materials and data acquisition

**Fig 1:**
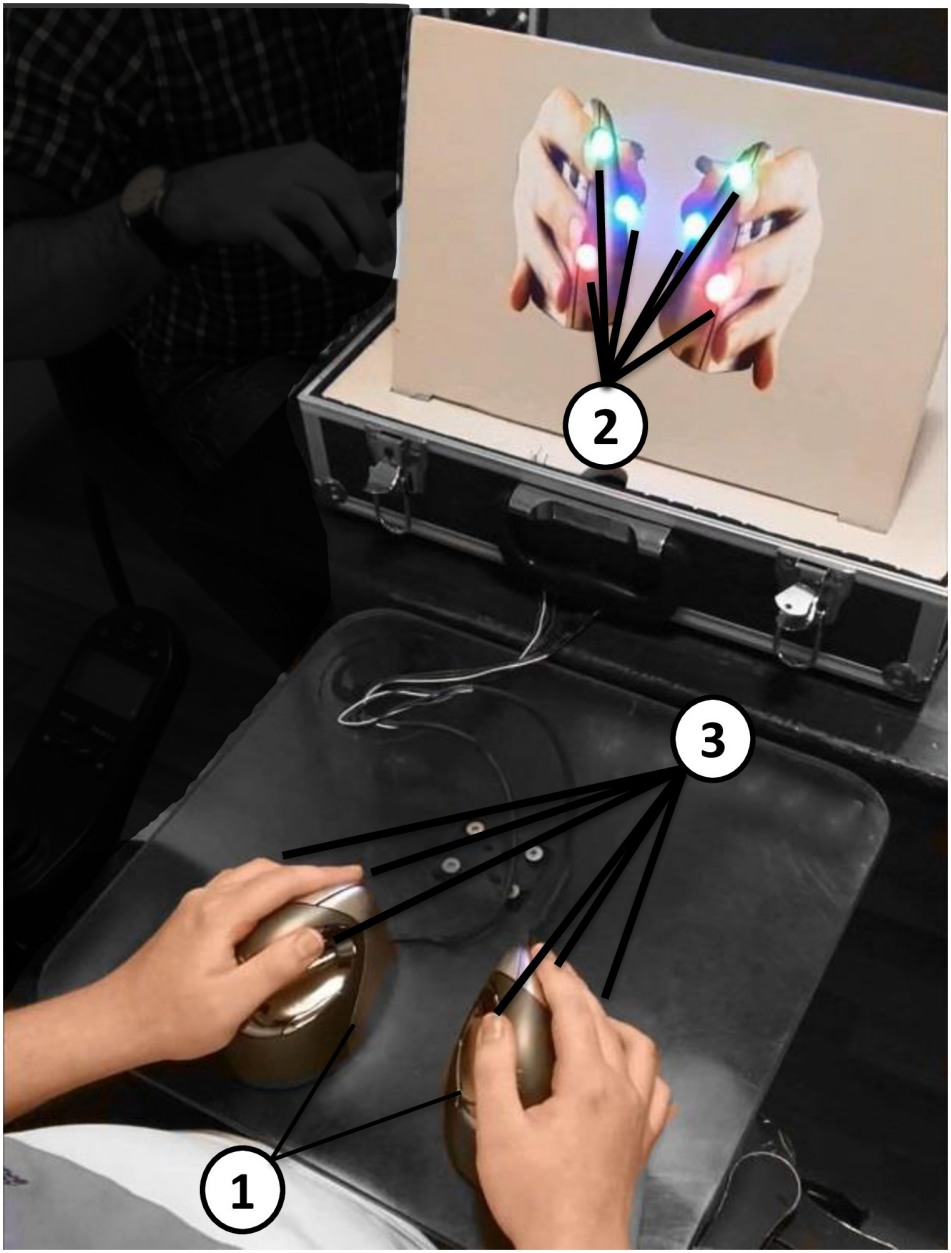
A participant with DMD, while using the portable setup with the proposed method, during the visuo-motor task (trial of 6 stimuli at 1 Hz). 1) Vertical mice with three buttons each. 2) LEDs: one for each index finger, middle finger, and thumb of each hand. The LEDs are also color coded: green for the index, red for the middle, and blue for the thumb; and 3) the number of fingers involved in this trial.

The setup (Fig 1) used for this experiment was developed at the University of Twente. It consists of a suitcase that contains all the components. The task consisted of a stimulus of blinking (ON/OFF) LEDs to which a participant had to respond by clicking mouse buttons that corresponded to LEDs that are ON. The LEDs were placed in a wooden board in front of the participant in an intuitive position (Fig 1). Two vertical mice, one right- and one left-handed, were used as an interface. The LEDs changed state synchronously over time with a frequency of 1, 2, …, 4 Hz, depending on the trial. The LEDs were ON or OFF with equal probability, independently of each other. The number of LEDs involved in a trial ranged from 1 to 6. The fingers involved were index, middle, and thumb of both hands. Performing all stimuli subsets for each of the four frequency steps results in a visuo-motor task with 24 different trials. After every trial, the perceived workload was verbally scored by the participants on a 1-20 scale [16]. The setup was chosen to enable the task for people with DMD and to resemble a game, since gaming can make the setup more familiar [17].

Based on previous studies on finger independence [18], finger involvement in functional grasps [19], and results on a grasp analysis questionnaire for people with DMD, we decided to include the thumb, index, and middle finger of both hands for the analysis of hand performance.

A real-time computer (myRio, National instruments Inc.) was used to control the visual stimuli for the participants. The same computer performed data acquisition, digitizing the mice signals at a sampling frequency of 24 Hz. All the data of the trials were logged. All electrical components were secured on the hollow part of the suitcase and protected by a wooden board. The LEDs and the mice had custom-made connectors, allowing for a quick set-up of the device to enhance its overall portability.

### Experimental procedure

The participants were placed in a chair in front of the setup (Fig 1). The protocol was explained to the participants, and they were allowed to become familiar with the device until they felt comfortable starting the experiment. The task included 24 trials, containing all combinations of four stimuli frequencies (1-4 Hz, with a step of 1 Hz) and simultaneous components (1-6 stimuli). The range of frequencies is sufficient to capture the limits of the human hand motor bandwidth [12], and the step of 1 Hz provides enough resolution, without making the experiment unnecessarily lengthy. We grouped the trials based on the number of simultaneous stimuli (six groups with four stimuli frequencies). To avoid order effects in our results, the group order was randomized per participant. Prior to each trial, the participant was informed about the frequency and the number of stimuli. After every trial, the participant was asked to score the perceived workload [16] on a visual analogue scale, where zero was a very low workload and 20 very high. This assessment technique was chosen because it is very simple to perform and reported as sensitive as multi-dimensional workload assessment techniques such as NASA-TLX [20]. Strong fatigue effects are often observed for people with DMD. Hence, for the participants with DMD, fatigue was also scored using an adjusted for the hand version of the 10-point OMNI scale of perceived exertion for walking/running [21]. The scale ranged from not tired at all (0) to very, very tired (10). The threshold we chose (2), corresponds to a little tired. If there was a reported score of above two, the participant took a short break (10 min), in order to make sure fatigue did not affect the results. Both scales were thoroughly explained to the participants, in order for them to understand that workload represents how mentally difficult each trial was perceived by the participants, while fatigue refers to how physically tired their fingers felt after each trial.

**Fig 2:**
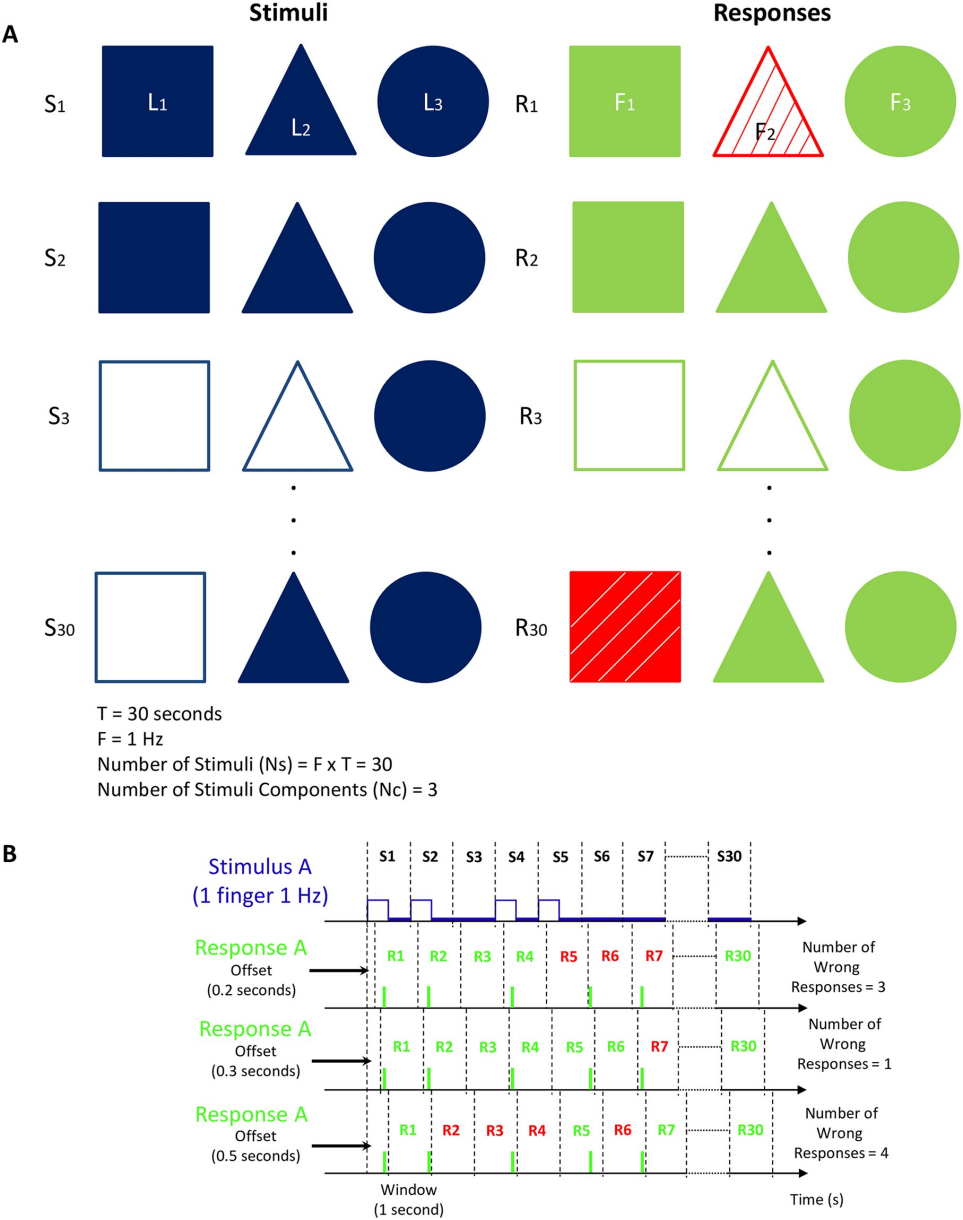
A) An example of a trial (three fingers at 1 Hz). In this example, we assume three LEDs (here shown as different shapes) blinking. Solid shapes mean LED on and click. Non-solid shapes mean LED off and no-click. As we have three LEDs for each stimulus, the responses come from three fingers responding to each LED. Red (with stripes) indicates a wrong and green (no stripes) a correct response. Note here, that a click when there is no stimulus provided is equally wrong to a no-click when a stimulus is provided. In this example, we assume 30 stimuli with a frequency of 1 Hz (1 stimulus/second). B) An example of the calculation of the offset for a trial of 1 finger with a frequency of 1 Hz, for one participant. Multiple values were applied for the offset, and the one which maximized the number of correct responses was chosen for the particular participant and trial (0.33 s in this example). The window size is the inverse of the trial frequency (1 second in this example) and the offset values are integer multiples of the sampling time (0.042).

Each trial had a duration of 30s, and, for each trial, a certain number of LEDs was used. This ensured that for the slowest trials (1 Hz), we have a minimum of 30 stimuli, a number sufficient for the estimation of the ITR, while keeping the time investment in the experiment low and the subject focused for the duration of each trial. For each stimulus in a trial, each LED involved was on or off with equal probability. The participants were instructed to click the button(s) based on the visual stimuli and to try to avoid random clicks. An example of a trial can be seen in Fig 2A. The order of finger recruitment is right and left index finger, right and left middle finger and right and left thumb, meaning that a trial including, for example, three fingers would be performed by the right and left index and the right middle finger. Healthy participants performed the task seven times over a period of three weeks, in intervals of three days. The total duration of the experiment varied from 35-60 minutes depending on the number of necessary breaks due to the fatigue of the participants with DMD.

### Data analysis

The first step in the data analysis was to determine for each component in each stimulus if the correct response was given. Note that each stimulus was offered for 1/*F* seconds, where *F* was the frequency of the trial. Every trial had a fixed duration of 30 s; therefore, the response signal per finger was divided into 30 ⋅ *F* adjacent intervals of 1/*F* seconds. We assumed that the response to a stimulus was given in the corresponding interval, which we will refer to as a window. The window is always the inverse of the frequency of a specific trial (for example the window is 0.5 seconds for a trial of 2 Hz). Since there was a response delay (incurred by both the participant and the experimental setup), we needed to offset the start of the windows. This offset was different for each participant and each trial, but it was taken as a constant within a trial. The offset was determined by computing for various values of the offset and the total number of correct responses in a trial. The offset was then fixed to the value that maximized the number of correct responses. A visualization of the window and offset for an example trial is illustrated in Fig 2B.

Every participant’s performance was assessed in terms of ITR and perceived workload. A brief explanation of the metrics is given in Table 1.

**Table 1:** Performance metrics.

Metric	Short Description
ITR (bits/sec)	The amount of mutual information between stimuli and responses [13].
Perceived Workload	Workload imposed by the visuo-motor task on the participants. It is assessed using a uni-dimensional assessment technique [16]

ITR is defined as the mutual information [22] between stimulus and response [12, 13]. We estimated the ITR per finger by counting the number of occurrences of each stimulus (on/off)—response (click/no click) pair. These numbers provided the maximum likelihood estimate of the probability of these pairs occurring in a trial and an estimate of ITR_finger_, the ITR per finger per stimulus, as

$$\begin{array}{c} \begin{array}{c} {\text{ITR}}_{\text{finger}}=\sum_{i\in \{\text{on},\text{off}\}}\sum_{j\in \{\text{noclick},\text{click}\}}I\left(n_{i},n_{j},n_{ij},N_{s}\right), \end{array} \end{array}$$ (1)

$$\begin{array}{c} \begin{array}{c} I\left(n_{i},n_{j},n_{ij},N_{s}\right)=\{\begin{array}{cc} \frac{n_{ij}}{N_{s}}{\text{log}}_{2}(\frac{n_{ij}N_{s}}{n_{i}n_{j}}), & \text{for}\;n_{ij}>0,\\ 0, & \text{otherwise}\;, \end{array} \end{array} \end{array}$$ (2)

$$\begin{array}{c} \begin{array}{c} {\text{ITR}}_{\text{est}}=F\cdot \sum_{1}^{N_{c}}{\text{ITR}}_{\text{finger}}, \end{array} \end{array}$$ (3)

where *n*_*ij*_ is the number of times event (*i*, *j*) occurs, *n*_*i*_ = ∑_*j*∈{noclick,click}_ *n*_*ij*_, *n*_*j*_ = ∑_*i*∈{on,off}_ *n*_*ij*_, *N*_s_ is the total number of stimuli provided in the trial and *N*_c_ the number of stimuli components. To illustrate, in a trial at 2 Hz, *N* = 30 ⋅ 2 = 60. The ITR_finger_ was summed over all fingers and multiplied with the frequency to obtain the total ITR_est_ in a trial, expressed in bits/sec. Note that, for the summation to be valid, we assumed that responses are independent across fingers. The ITR of a response that is completely unrelated to the stimulus is zero. This implies that random clicking (as would occur, for instance, in a trial that is too difficult) results in zero ITR. In this sense the ITR is a more appropriate measure than the success probability of providing the correct response which would be 50% for random clicking. Additionally, we analyzed the normalized ITR (ratio of ITR, to provided information per trial).

The provided information (PI) per trial refers to the amount of bits/sec that we provide the participant via the visual stimuli. This was calculated as:

$$\begin{array}{c} \begin{array}{c} PI_{\text{trial}}=F\cdot N_{c}. \end{array} \end{array}$$ (4)

### Statistical analysis

All statistical tests were performed depending on whether the groups we wanted to compare were normally distributed or not and whether they were independent or paired. To check for the normality assumption, we used the Shapiro-Wilk test. All the data used for the statistical analysis together with the fatigue scale scores and the offset values used for the first session of the healthy and participants with DMD, will be available online as a complementary file to this article.

To compare ITR, normalized ITR and workload between healthy and DMD participants, we performed the non-parametric Mann-Whitney test for independent samples (Shapiro-Wilk test: *p* < 0.005).

Furthermore, we wanted to compare healthy and DMD participants for each trial. To do this, we treat the ITRs of our healthy participants as observations from a “healthy” population that was normally distributed with a mean and a variance different per trial. For each trial and each DMD participant, we considered the null hypothesis that the ITR of the DMD participant was an observation from the ITRs of the healthy population. Based on this, we computed the lower-tailed *p*-value, corresponding to the test that the ITR of the DMD participant was significantly smaller than the ITR of healthy participants. The computation of the *p*-value was done according to [23], i.e. specifically for the situation that we were comparing a single case with a control sample.

For the assessment of training effects on ITR and workload, the non-parametric Wilcoxon signed-rank test for dependent samples was used (Shapiro-Wilk test: *p* < 0.05).

The statistical tests were performed with SPSS (IBM SPSS Statistics 24).

Additionally, we performed a Pareto optimization analysis to illustrate the trade-off between ITR and workload. The trials in which the ITR cannot be improved without increasing the workload are called Pareto optimal [24]. We used this analysis to compare healthy and DMD participants as well as to visualize the effects of training.

## Results

After a first analysis of the recorded data, we noticed that trials of 4 Hz showed very small ITR. This was probably because the participants were not able to cope with these trials in a meaningful way, and mostly random clicking was observed. Therefore, we decided to exclude the 4 Hz trials from the data analysis and the visualization of the results.

### Healthy vs. DMD

**Fig 3:**
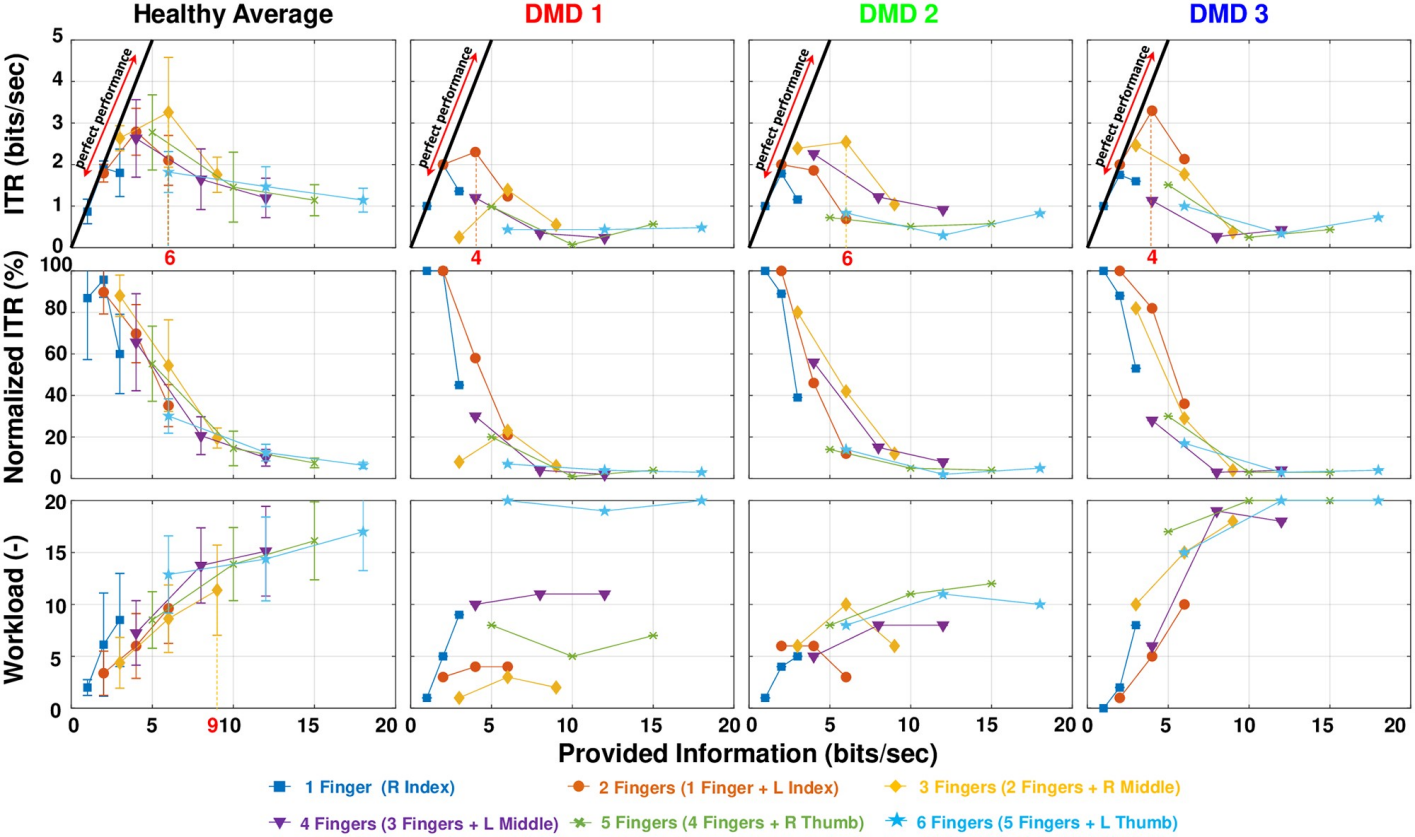
The results for all participants. The top four graphs show the raw values of ITR in bits/sec. The middle plots show the normalized ITR and the bottom the perceived workload of all the subjects. For the healthy group, average mean and standard deviation are plotted for each trial. Provided information can depict more than one trial at the same point. For example (top left plot), 6 bits/sec can represent the second yellow data point (three fingers ⋅ 2 Hz), the third orange data point (two fingers ⋅ 3 Hz)and the first light blue data point(6 fingers ⋅ 1 Hz). The diagonal line represents perfect performance (where provided information is equal to ITR). The DMD participants are placed in order of age from the youngest to the oldest.

**Fig 4:**
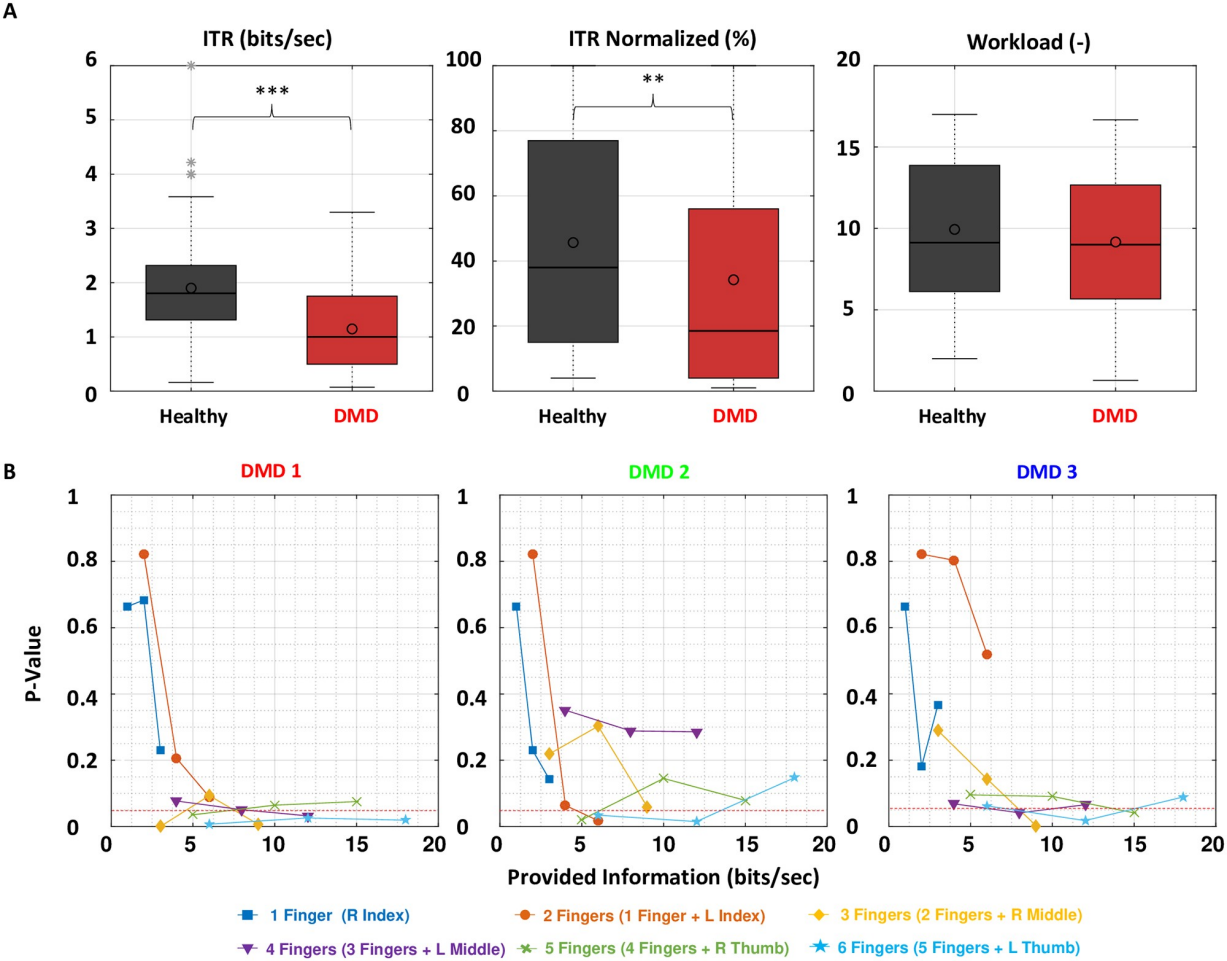
A) Box plots of ITR, normalized ITR and workload during the first session of the healthy group and the DMD participants for all trials. Horizontal lines represent the median while circles represent the mean values. * indicates a significant difference at the level of p<0.05, ** indicates p<0.01 and *** indicates p<0.001. B)The differences between the healthy and every DMD participant per trial, regarding ITR.

Fig 3 shows the results of ITR and workload as a function of the provided information for all participants. Fig 4A summarizes, together with Table 2, the statistical analysis performed on each metric between the healthy and DMD participants. Fig 4B shows a per trial comparison between healthy and DMD participants for ITR. Fig 6A shows for all participants the optimal trials found from the Pareto analysis.

**Table 2:** Summary of the means with standard deviations and the statistical tests.

Metric/Participant		Healthy				DMD		
ITR (bits/sec)	Mean(±std)	1.9(±0.88)				1.14(±0.75)		
	Mann-Whitney test	**p < 0.001**						
Normalized ITR (%)	Mean(±std)	46(±34)				34(±36)		
	Mann-Whitney test	**p = 0.004**						
Workload (-)	Mean(±std)	9.9(±5.56)				9.1(±6.15)		
	Mann-Whitney test	p = 0.303						
**Metric/Session**		**1**	**2**	**3**	**4**	**5**	**6**	**7**
ITR (bits/sec)	Mean(±std)	1.9(±0.88)	2.3(±1.04)	2.5(±1.21)	2.7(±1.31)	2.8(±1.41)	2.9(±1.48)	3.0(±1.53)
	**Comparisons**	1-2	2-3	3-4	4-5	5-6	6-7	-
	Wilcoxon signed-rank test	**p < 0.001**	**p = 0.007**	**p = 0.013**	**p = 0.007**	**p = 0.014**	p = 0.094	-
Normalized ITR (%)	Mean(±std)	1.9(±0.67)	2.3(±0.81)	2.5(±0.92)	2.7(±1.03)	2.8(±1.16)	2.9(±1.15)	3.0(±1.12)
	**Comparisons**	1-2	2-3	3-4	4-5	5-6	6-7	-
	Wilcoxon signed-rank test	**p < 0.001**	p = 0.084	**p = 0.014**	**p = 0.009**	p = 0.161	p = 0.173	-
Workload (-)	Mean(±std)	9.9(±5.56)	8.9(±6.03)	8.3(±5.86)	7.6(±5.40)	7.2(±5.16)	7.2(±5.28)	6.6(±5.18)
	**Comparisons**	1-2	2-3	3-4	4-5	5-6	6-7	-
	Wilcoxon signed-rank test	**p = 0.001**	p = 0.075	**p = 0.002**	p = 0.05	*p* = 0.895	**p = 0.005**	-

Bold *p*-values indicate a significant difference at the *p* = 0.05 level.

#### ITR

The highest mean ITR value for the healthy participants was achieved for the three fingers-2 Hz trial. DMD 1 achieved a maximum ITR at two fingers-2 Hz trial, yet with only 60% of the maximum ITR he could achieved in this trial. His maximum ITR was the lowest achieved among the DMD participants (2.30 bits/sec). DMD 2 achieved a maximum ITR for three fingers-2 Hz (80% of the maximum achievable in this trial), similar to the healthy average and DMD 3 achieved maximum for the two fingers-2 Hz trial. His maximum (3.29 bits/sec, 81% of the maximum achievable in the trial) was the highest value among the DMD participants as well as higher than the mean ITR of the healthy participants (3.25 bits/sec and 90% of the maximum achievable for this trial). We can see that healthy participants and DMD 2 achieved the highest ITR at 6 bits/sec of provided information while DMD 1 and DMD 3 at 4 bits/sec. Regarding the normalized ITR, healthy participants achieve on average values > 20% for more than four fingers and for five and six fingers at 1Hz. The decline in percentage appears to be smoother in the healthy participants than in participants with DMD, especially when more than three fingers are tested (even two in the case of DMD1).

A difference was identified for ITR (Mann-Whitney test, *p* < 0.001) and normalized ITR (Mann-Whitney test, *p* = 0.004) between healthy and DMD participants. A comparison per trial (Fig 4B) revealed that the difference between healthy participants and participants with DMD increases when more fingers are involved.

#### Workload

For workload, no statistically significant difference was found between healthy and DMD participants (Mann-Whitney test, *p* = 0.303). Average scores appeared to be slightly lower for DMD 1 and 2, whereas DMD 3 scored slightly higher (Fig 3). Trends were comparable to the healthy group for all DMD participants. Generally, 3 Hz trials with large number of fingers were experienced the hardest, whereas the 1 Hz trials (with low number of fingers) were experienced as the easiest. However, DMD 1 showed a different workload pattern from all the other participants.

#### Pareto analysis

**Fig 5:**
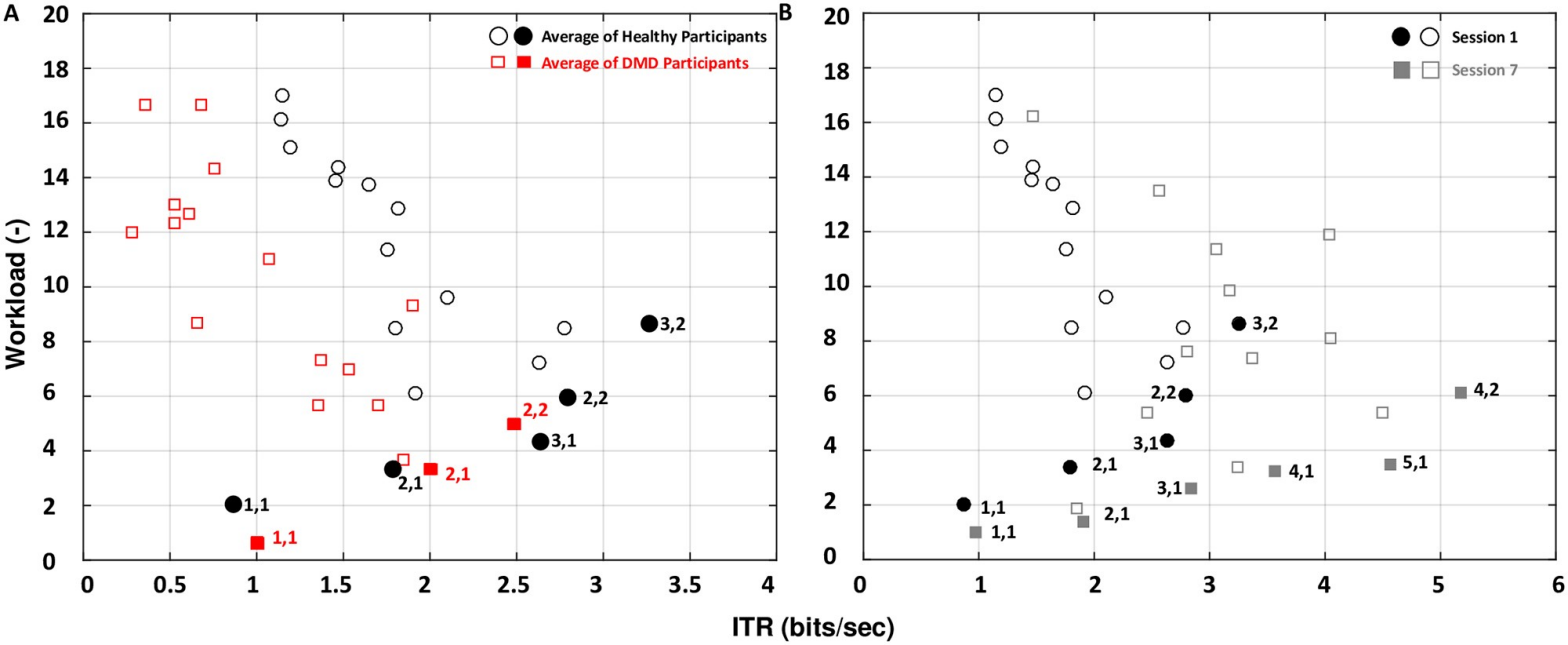
The trade-off between ITR and workload. The first number is the number of fingers and the second is the frequency. Solid shapes indicate Pareto optimal trials. Optimal is a trial from which we cannot go to higher ITR without also raising the workload. (A) Pareto optimal trials of healthy participants for the first session together with the optimal trials of DMD participant, and (B) the shift in Pareto optimal trials after seven sessions for the healthy participants.

The Pareto analysis (Fig 5A) shows the optimal trials with respect to the trade-off between ITR and workload. Optimal is a trial from which we cannot go to higher ITR without also raising the workload. Healthy and DMD participants showed similar performance. The trials of one finger at 1 Hz and two fingers at 1 and 2 Hz were common optimal trials among healthy and DMD participants. However, healthy participants also showed optimal trials for three fingers at 1 and 2 Hz.

### Training

**Fig 6:**
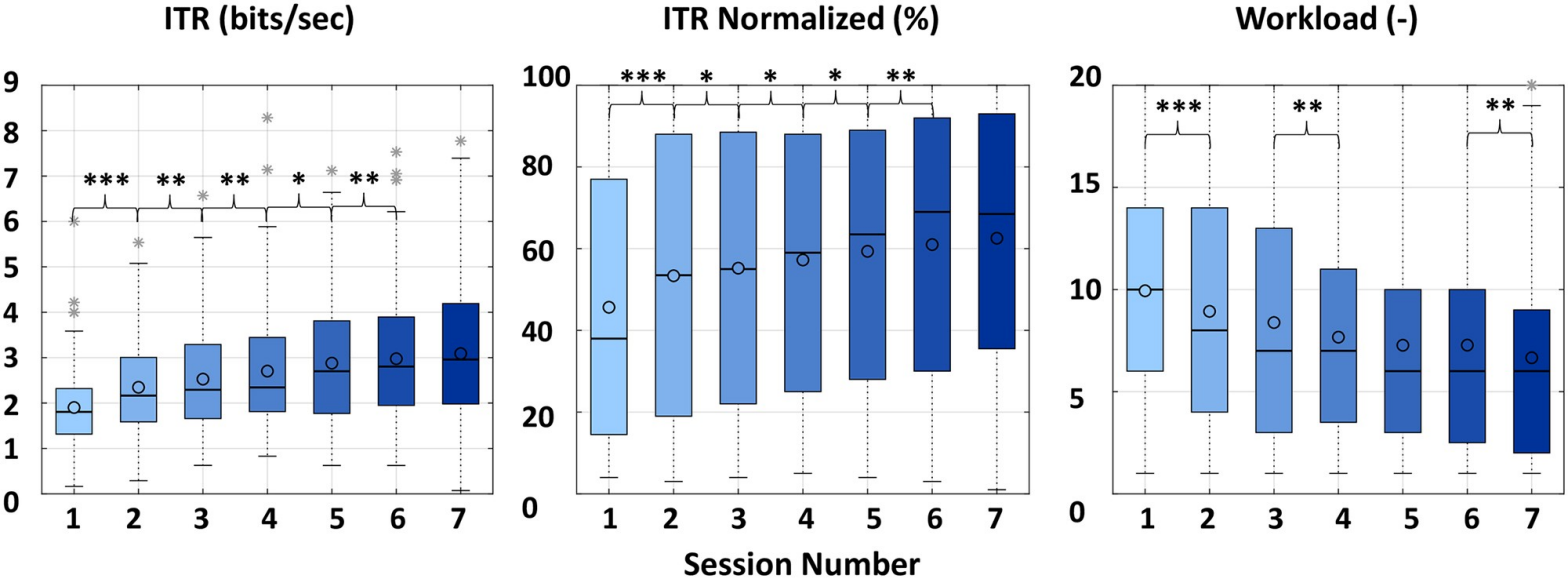
Box plots of ITR, normalized ITR and workload during the training period of seven sessions for the healthy participants. Horizontal lines represent the median while circles represent the mean values. * indicates significant difference at the level of p<0.05, ** indicates p<0.01, and *** indicates p<0.001.

Fig 6 and Table 2 summarize the results of the statistical analysis performed in each metric to show the results of training. Fig 5B shows the change in the optimal trials due to training. There were significant improvements between the seven sessions for each metric, suggesting a learning effect.

#### ITR and workload

Wilcoxon signed-rank test showed a significant improvement for ITR between sessions 1 and 2 (*p* < 0.001), 2 and 3 (*p* = 0.007), 3 and 4 (*p* = 0.007), 4 and 5 (*p* = 0.013) and 5 and 6 (*p* = 0.004). The participants reached their highest mean ITR in session 7 (ITR = 3.0 ± 1.53 bits/sec), without significant improvement from session 6 (*p* = 0.094). For the normalized ITR the same learning effect was observed. There was again significant improvement between sessions 1 and 2 (*p* < 0.001), 2 and 3 (*p* = 0.011), 3 and 4 (*p* = 0.042), 4 and 5 (*p* = 0.034) and 5 and 6 (*p* = 0.008). The participants reached their highest mean normalized ITR in session 7 (normalized ITR = 61 ± 33%), without significant improvement from session 6 (*p* = 0.126). Workload was also significantly different between sessions 1 and 2 (*p* = 0.001), 3 and 4 (*p* = 0.002) and 6 and 7 (*p* = 0.005). The lowest mean workload was observed for the 7th session (6.6 ± 5.18).

#### Pareto analysis

In Fig 5B, we can see that, after seven sessions, different trials became optimal. These were trials with four fingers at 1 and 2 Hz (4,1 and 4,2) and five fingers at 1 Hz (5,1). The trials with two fingers and three fingers at 2 Hz were no more optimal after the training was completed and the number of optimal trials increased from five to six.

## Discussion

### Number of participants and protocol

Due to the low density of the DMD population, we aimed to include only three participants. This choice was motivated by the exploratory nature of this work and our commitment to cause the least possible inconvenience to our target population, taking in to account that they participate in multiple studies. Therefore, our conclusions must be regarded with caution.

People with DMD often experience early fatigue onset. Therefore, we took this into account and developed a protocol ensuring that enough trials were performed (24 trials, with a bandwidth of 4Hz and duration of 30 seconds) with the appropriate variability for extracting useful conclusions while keeping the experiment short in time and giving enough breaks to the participants, in order to keep participant burden low and focus high. Additionally, we monitored their fatigue throughout the experiment and offered them breaks when needed.

The results regarding task performance reported in this study cannot be attributed to motor performance alone. With the introduction of the workload evaluation after every trial, we aimed to capture the cognitive effort and analyze it together with motor performance. The workload score used in this study, exhibits superior sensitivity compared to other common workload assessment techniques and it is ideal as a screening tool for identifying workload bottlenecks [16]. One limitation of this score however, is the fact that it is subjective and may be affected by the randomized order of the trials. However, all participants were allowed to familiarize themselves with the task and different randomization of orders was applied to all participants. Therefore, we believe that the effect on the workload scale was similar for both healthy and DMD participants, so comparison of the two groups is valid. Regarding our participants, DMD 1 had inconsistent and unexpected workload scores (for example, all five finger trials scored lower than four finger trials). This may indicate that he suffers from cognitive deficits, which may partly explain the lower task performance that we found for this participant. However, in this study we did not explicitly assessed the cognitive profile of our participants.

### Healthy vs. DMD

We found a significant difference in task performance between healthy and DMD, only for ITR, as there was no statistically significant difference in subjective perceived workload in this study between healthy and DMD participants (Fig 4A and Table 2). The Pareto analysis revealed similarities for trials of one and two fingers. However, healthy people had optimal trials also for three fingers. By analyzing the ITR per trial, we found also that those similarities in task performance are mainly for one up to three fingers. Recent studies with people with DMD have shown that no use of their limbs is disuse [25], resulting in physical impairments like contractures, affecting their motor performance. Therefore, we believe that the disuse of their fingers probably made it difficult for them to retain performance similar to healthy people for more than three fingers, despite the fact that the employed visuomotor task required minimal finger movement.

### Training

In order to determine if and where there can be any improvement on the experimental task, we performed a training measurement consisting of seven sessions. The motivation behind these training measurements was to see if the ITR values reported in the first session are the limits of human hand task-related cognitive-motor performance, or whether they are subject to further improvement. The order of trials was randomized differently for each participant, ensuring that there are no order effects in the improvement found in training. We observed significant improvement in both metrics, indicating better motor performance and lower cognitive effort as a result of a short training period. There are two ways to increase ITR. One is by increasing the number of stimuli components and the other by increasing the stimuli frequency. According to our results, participants were able to improve the number of stimuli components (fingers) that could (successfully) be processed, rather than an increase in frequency. We found that our healthy participants, after training, reached the maximum ITR scores at the frequency of 2 Hz. This corresponds to the results of Klemmer et al. wherein the highest ITR values were at 2.4 Hz [13].

Keeping the limitations of people with DMD in mind, we did not perform a training study with them, in order to cause the least inconvenience possible to our participants. However, we believe that, despite the progressive deterioration of their hand function, individuals who retain some functionality may also improve in a time frame of three weeks, since motor learning does not change as a result of the disease [26]. A recent study showed that people with DMD experience even stronger training effects than healthy controls during a computer task, and they are able to acquire and retain motor learning improvements after training [27]. Hence, we assume improvement of their motor performance, similar to what we observed for healthy people. However, the visuo-motor task used in our study requires substantial cognitive processing. This can be seen in the Pareto analysis, where trials that require a lower cognitive effort yield higher task performance. People with DMD mainly experience motor impairments (primarily muscle weakness), but a large number of people with DMD also experience cognitive impairments and impaired ability in processing visual information [28]. A recent study showed that even individuals with DMD without intellectual disability have a deficit in implicit learning [29]. Therefore, we cannot assume that individuals with DMD will also improve due to implicit learning that will lead to lower cognitive effort.

### Relevance

The results of this study are particularly relevant given the lack of analyses concerning the cognitive-motor hand performance of this specific population. They are also relevant given the expected increase in DMD population and the related need to introduce customized hand rehabilitation for people with DMD [5] and a multidisciplinary approach to create preventive measures and interventions for the rehabilitation of people with DMD [3, 30].

As suggested by Wagner et al. [9] and as shown by Weichbrodt et al. [8], rehabilitation for people with DMD should aim for the retardation of the progress of the disease and the preservation of certain motor performance. Currently, passive stretching of muscles [5] and resting hand splints during sleep [8] are clinically used for the hand rehabilitation of people with DMD. In 2010, Bushby et al. [30] proposed a new set of care guidelines with additional therapeutic options for multi-disciplinary hand rehabilitation of the DMD population. Those aim to extend traditional rehabilitation with the use of technology [31, 32]. They also suggest the use of active devices. The development of such a device is the project goal of the Flextension Symbionics project [11].

Based on the current training results of the healthy controls, we expect that people with DMD may also improve their motor-related performance of four and five fingers already within a few weeks. Additionally, the differences in task motor performance indicates that healthy adults are able to achieve higher cognitive-motor performance related to the control of the hand, especially for more than three fingers. Despite the fact that the employed visuo-motor task is not resembling daily activities involving finger motion, this difference may be linked to disuse of fingers in the DMD population [25] and to the modest results of current hand rehabilitation. Therefore, we believe, in line with the recommendation by Bushby et al. [3, 30] and Jansen et al. [25], that early multi-disciplinary rehabilitation of people with DMD should involve dynamic multi-finger rehabilitation and promote use, in order to help them retain a higher cognitive-motor hand performance. This can be complemented by active-hand devices for home rehabilitation in combination with gaming, similar to what was done by Amirabdollahian et al. [33] for stroke rehabilitation. In this way, the user will actively participate in the rehabilitation process and may be motivated to use his own fingers more. Additionally, more effective hand rehabilitation can enable the use of sophisticated hand orthoses, which can provide multi-finger daily assistance to individuals with DMD.

Future work will include more participants with DMD from various age groups in order to capture the hand cognitive-motor performance of people with different progression levels of DMD. Additionally, a more extensive protocol including a cognitive profile assessment of the participants prior to the task will help to analyze further the relationship between cognitive and motor performance in task assessment and training. Lastly, it is interesting to explore the effect of training in people with DMD, yet without encumbering them and keeping in mind the early fatigue onset they often experience (i.e. use a modified training protocol). Nonetheless, the presented protocol was able to offer a systematic analysis of their task-related hand motor-cognitive performance and highlight the relevance of hand rehabilitation in DMD.

## Conclusion

We compared the cognitive-motor performance of healthy people and people with DMD during a hand-related visuo-motor task and analyzed this together with their perceived workload. We also studied the training effects related to the repeated application of our protocol. People with DMD showed an overall lower task performance compared to the healthy controls. However, this was mainly seen when more than three fingers were involved. Both metrics presented improvement in healthy persons when training was provided. However, training mainly led to improvement over the number of fingers involved rather than the frequency. Early hand rehabilitation of people with DMD may include dynamic multi-finger training to help, together with the use of active assistive devices, to reduce finger disuse and improve hand related cognitive-motor performance. We believe that early hand rehabilitation of people with DMD should focus on dynamic multi-finger training. This may help, together with the use of active-assistive devices, to reduce finger disuse and improve hand-related cognitive-motor performance. Regardless of the low number of participants with DMD included in this study, we gained useful insights into the cognitive-motor hand performance of people with DMD, related to our task. In order to generalize our results, additional research with more people with DMD is needed. In the future, we will apply our conclusions in the design of engaging and customized hand rehabilitation for people with DMD, in combination with an active-hand-assistive device.

## Supporting information

S1 File Experimental data file with all metric values for the eight healthy and the three DMD participants. This file includes the values of ITR (raw and normalized) and workload for all participants in this study. Additionally, it contains the offset values for all participants and all trials and the fatigue scores of the participants with DMD.
(XLSX)

S1 Fig (PDF)
