# Supplementary material for: Evaluation of the cognitive-motor performance of adults with Duchenne Muscular Dystrophy in a hand-related task
Source: PLoS One. 2020 Jan 31;15(1):e0228128. doi: 10.1371/journal.pone.0228128 (PMC6993979; doi:10.1371/journal.pone.0228128)
Supplement: S1 Fig — (PDF) [file pone.0228128.s002.pdf]

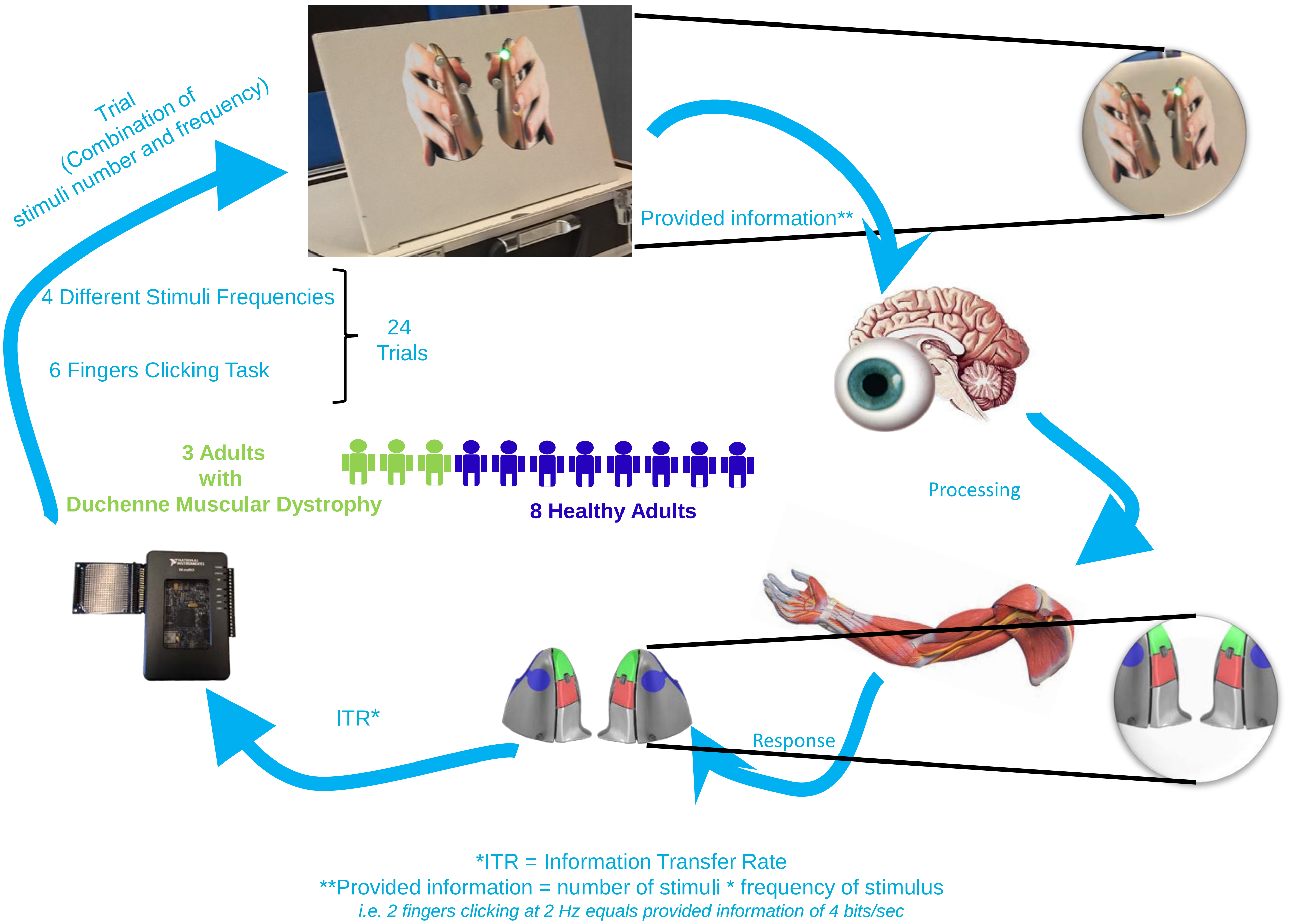
Trial
(Combination of
stimuli number and frequency)
Provided information**
4 Different Stimuli Frequencies
6 Fingers Clicking Task
24
Trials
3 Adults
with
Duchenne Muscular Dystrophy
8 Healthy Adults
Processing
ITR*
Response
*ITR = Information Transfer Rate
**Provided information = number of stimuli * frequency of stimulus
i.e. 2 fingers clicking at 2 Hz equals provided information of 4 bits/sec
